# Eugenics and Sentiment

**Published:** 1913-10

**Authors:** 


### BUFFALO MEDICAL JOURNAL

**A Monthly Review of Medicine and Surgery**

EDITOR AND PUBLISHER DR. A. L. BENEDICT, 228 Summer St., corner of Elmwood Ave., Buffalo. (Address for all communications. Please make personal and telephone calls before 1 P. M.)

THE BUFFALO MEDICAL JOURNAL is the third, in age, in the western continent. It is independent but supports professional organization. As a scientific medium it is unrestricted in territory. As a news medium, it is limited mainly to the western four districts of the Medical Society of the State of New York. The co-operation of physicians is asked in securing personal, obituary and other news items. Secretaries of Medical Societies in this region are requested to send brief reports of meetings. Full transactions will be published at cost of composition.

Subscription $2.00 a year in advance; $3.00 for two years in advance. Changes and errors in address or failure or duplication of delivery, should be reported immediately. Back numbers will be supplied if possible but requests for copies should be made promptly,

YEARLY VOLUME 69 OCTOBER, 1913 No. 3

### Eugenics and Sentiment

Sentiment is justly regarded as inappropriate to medical journalism, but certain aspects of the modern campaign for eugenic marriages suggest that there may be a practical side to sentimental views. First of all, we advocate strongly the old-fashioned view that the basis of marriage ought to be love, meaning by love neither lust nor cold-blooded esteem, but a decent, hearty, sex-attraction strongly individualized. How, further, to reduce love to a schedule and to decide in advance whether it is adequate to withstand the trials of future married life, we do not know. We have seen disastrous results from long engagements, founded on previous friendship and every apparent favorable circumstance as to family acquaintance, heredity, community of tastes and social standing, and we have seen ideal marriages after elopements or very short acquaintance and with every conceivable difference of taste and environment that ought theoretically to have caused trouble. And, to be fair, we must concede that one of the most fortunate marriages that we have observed, followed a perfectly business-like search for a wife able to support a husband in the style to which neither he nor his family had been accustomed for several generations.

Even from a cold-blooded standpoint, it must be conceded that love, as a basis for marriage, is not entirely unscientific. The influence of psychic states on physical phenomena is supported by good authority. In particular, we know that digestion and nutrition depend very largely upon appetite, so much so that considerable differences in nutritive value and theoretic digestibility must be disregarded in selecting foods. The very fact that the analogy to reproductive processes has been so widely and so coarsely recognized is the best evidence that the analogy, as applied to eugenics, is a genuine one.

While marriage must be frankly recognized as a sex-relation, various social institutions and, most important of all, the maintenance of marriage and proper support of and care for the rising generation, demand that the idealized conception of sex, gradually developed in the evolution of man to a state of enlightenment, must be maintained. The family cannot be preserved if conventional developments are so radically changed that the differences between men and women are reduced to their original anatomic and physiologic basis. This statement, however, should not be construed to represent a reactionary antagonism to all forms of progress and of adaptation of customs to modern requirements. While the editor acknowledges a considerable degree of conservatism in regard to the modern extension of women's activities, care must be taken not to confuse prejudice with principle.

There is one important item in which conventionalities in regard to sex are bound soon to raise an issue. Very properly, the medical profession and sociologists generally are advocating medical examination as a prerequisite to marriage. Such examination is already legally required by some states and the requirement will undoubtedly soon become quite general. But, if we stop to consider the matter, not in the abstract but in the very concrete form in which it will be presented to individual engaged couples and their families, it will become evident that the literal application of the requirement to young women is decidedly objectionable from the conventional standpoint and the examination will, undoubtedly, be reduced to a rather nominal degree. Unfortunately, too, as all of us know from individual acquaintance, there are instances in which such examination is desirable even from the same crude standpoint frankly admitted for men. Hence, the legal requirement of an examination cannot fail to produce, in a small but appreciable percentage of cases, a sense of false security and to lead to recriminations, especially of the examiner. Only recently we have noted the marriage of very attractive but decidedly damaged goods, and it is perfectly possible that the damage may include actual danger from the standpoint of eugenics. We have also been impressed with the frequency with which widows, of either kind, have, without personal blame or realization, visited the sins of one husband upon the offspring of the successor. Yet, with all allowance for such unfortunate cases, it is very questionable whether the ultimate results of a thorough physical examination of all prospective brides would not exceed, in indirect social disadvantage, the direct benefits of detecting disease of various kinds.

It must not be forgotten that, while eugenics has recently assumed an unprecedented quantitative importance in medical and sociologic literature, it is by no means a new subject nor one on which the laity are entirely uninstructed. It is true that there has been a gap of many years between empiric and modern scientific eugenic legislation, but the realization of the general principles has been very wide spread and, to a surprising degree, put into actual practice. Every physician can recall necropsies requested to settle the question of cancerous heredity, in the interests of sons and daughters of marriageable age, of engagements broken because of tuberculosis or merely because one or other party was "sickly," of young men soliciting gonococcus tests. There is urgent need, not so much of popular instruction as of accurate tests of ideas as to heredity held by the profession, to insure against the teaching of erroneous doctrine.

